# The added prognostic values of baseline PET dissemination parameter in patients with angioimmunoblastic T‐cell lymphoma

**DOI:** 10.1002/jha2.610

**Published:** 2022-11-29

**Authors:** Huanyu Gong, Bo Tang, Tiannv Li, Jianyong Li, Lijun Tang, Chongyang Ding

**Affiliations:** ^1^ Department of Nuclear Medicine Jiangsu Province Hospital The First Affiliated Hospital of Nanjing Medical University Nanjing China; ^2^ Department of Radiology Shuyang Hospital of Traditional Chinese Medicine Suqian China; ^3^ Department of Hematology Jiangsu Province Hospital The First Affiliated Hospital of Nanjing Medical University Nanjing China

**Keywords:** angioimmunoblastic T‐cell lymphoma, dissemination, FDG, positron emission tomography, prognosis

## Abstract

To explore the prognostic values of baseline 2‐deoxy‐2‐[^18^F] fluoro‐D‐glucose (FDG) positron emission tomography/computed tomography (PET/CT) dissemination parameter in angioimmunoblastic T‐cell lymphoma (AITL) and its added values to total metabolic tumour volume (TMTV). Eighty‐one AITL patients with at least two FDG‐avid lesions in baseline PET/CT were retrospectively included. PET parameters including TMTV and the distance between the two lesions that are the furthest apart (Dmax) were obtained. Univariate Cox analysis showed that both Dmax and TMTV were risk factors for progression‐free survival (PFS) and overall survival (OS). Multivariate Cox analysis models of different combinations showed that high Dmax (> 65.7 cm) could independently predict both PFS and OS, while high TMTV (>456.6 cm^3^) was only significant for OS. A concise PET model based on TMTV and Dmax can effectively risk‐stratify patients. PFS and OS rates were significantly lower in patients with high Dmax and high TMTV than in patients with low Dmax and low TMTV (3‐year PFS rate: 15.0% vs. 48.7%, *p* = 0.001; 3‐year OS rate: 27.6% vs. 79.0%, *p* < 0.001). Dmax can directly reflect the disease dissemination characteristic and has a significant prognostic value for FDG‐avid AITL patients. It has the potential to be introduced into new risk stratification models for tailored treatment.

## 1 INTRODUCTION

Angioimmunoblastic T‐cell lymphoma (AITL) is a rare hematologic malignancy originating from mature T follicular helper cells and is the second most common pathologic subtype of peripheral T‐cell lymphoma (PTCL), accounting for approximately 15%–20% of PTCL and 1%–2% of non‐Hodgkin's lymphoma (HL) [1, 2]. AITL is typically characterized by an aggressive course, progressive generalized lymphadenopathy, B symptoms, hepatosplenomegaly and the advanced Ann Arbor stage [3]. This malignancy is prone to progression and has a generally dismal prognosis, with 5‐year overall survival (OS) rate ranging from 30% to 40% [4, 5]. Therefore, it is important to identify those at high risk for better management.

The International Prognostic Index (IPI) and the Prognostic Index for T‐cell lymphoma (PIT) are commonly used as risk assessment methods in AITL patients during clinical practice, but both are non‐specific indicators with limited efficacy for risk stratification [6]. The Prognostic Index for AITL(PIAI) and the AITL score are the AITL‐related prognostic models based on independent AITL cohorts [4, 5]. Recently, the latter is considered to better reflect the characteristics of AITL and allows for more effective risk stratification than the other three models. But, all of the above models were established based on clinical parameters without reliable tumour burden parameters. The value of 2‐deoxy‐2‐[^18^F] fluoro‐D‐glucose (FDG) positron emission tomography/computed tomography (PET/CT) in the management of lymphoma has been confirmed in recent years. As a quantitative PET parameter, total metabolic tumour volume (TMTV) is currently considered to indicate tumour burden in FDG‐avid lymphoma. Some publications show that high baseline TMTV may be an independent prognostic factor for PTCL, including AITL [7, 8]. Baseline TMTV combined with clinical models or interim PET (iPET) responses can make favourable prognostic risk stratification for PTCL patients [9, 10, 11].

The concept of the distance between the two lesions that are the furthest apart (Dmax) was first proposed by Cottereau et al [12]. Dmax, as a new PET parameter, reveals the tumour dissemination and invasive ability from another dimension and has shown added prognostic value in HL and diffuse large B‐cell lymphoma (DLBCL) [13, 14, 15, 16]. Besides, TMTV combined with dissemination parameters could be superior to IPI for risk stratification of DLBCL patients [14]. However, publications about Dmax in AITL were rare, and the main objective of the present study was to preliminary investigate the prognostic value of Dmax in AITL.

## 2 MATERIAL AND METHODS

### 2.1 Patients

**FIGURE 1:**
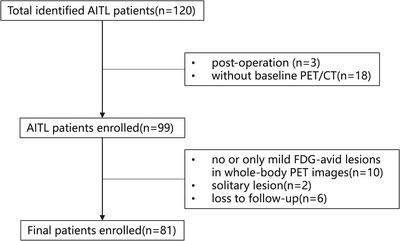
The flowchart of inclusion and exclusion process for the whole group of patients

Patients with the histologically confirmed diagnosis of AITL who underwent pretreatment ^18^F‐FDG PET/CT between April 2009 and October 2021 were retrospectively collected. To accurately calculate Dmax and TMTV, we excluded patients with no or only mild FDG‐avid lesions in PET images. In addition, patients with only a solitary lesion were also excluded. The process of inclusion and exclusion is shown in Figure 1. Clinical parameters were retrospectively collected from the medical record system, including clinical characters (age, gender, Ann Arbor stage, Eastern Cooperative Oncology Group performance status [ECOG‐PS] score, B symptoms, site of extranodal involvement [ENIs]), laboratory test data (bone marrow biopsy, haemoglobin, platelets [PLT] counts, lactate dehydrogenase [LDH], β2‐microglobulin (β2‐MG), albumin, C‐reactive protein (CRP), the copy number of Epstein‐Barr Virus DNA (EBV‐DNA)] and treatment regimens. This study complies with the Declaration of Helsinki. All informed consent forms were waived because this study was retrospective. It was approved by the Ethics Committee of Jiangsu Province Hospital, the First Affiliated Hospital of Nanjing Medical University.

### 2.2 PET/CT image analysis

The PET/CT scanning protocol was consistent with the previously published study [8]. Image interpretation was carried out by two experienced nuclear medicine diagnostic physicians blinded to the patient's clinical outcome. When in doubt, the results were determined by a consensus between the two physicians. MTV was delineated semi‐automatically by ‘the petctviewer plugin in Fiji software’ with the 41% maximum standardized uptake value method and calculated (manually removing physiological regions and non‐lymphoma lesions). The oropharyngeal regions with only high FDG uptake but no positive CT findings were excluded from MTV calculations. TMTV was the sum of MTV for all voxels of interest. The criteria of bone marrow involvement (BMI) were positive bone marrow biopsy or focal high uptake on PET. Spleen involvement was considered if the spleen vertical diameter was >13 cm or the spleen uptake was higher than normal liver background uptake [17]. The 3D coordinates of each lymphoma lesion, including nodal and extranodal sites, were obtained using LIFEx software (version 7.20, https://www.lifexsoft.org) according to the previous literature [18]. The centre of each lesion (centroid) was adopted as the location of the corresponding lesion, and Dmax was calculated by the Euclidian formula [12].

### 2.3 Risk assessment methods and surveillance

Four clinical methods were utilized for risk stratification in this study, including the IPI, PIT, PIAI and the AITL score, respectively (Table S1). In this study, the high‐risk patients were defined as IPI > 2, PIT > 2, PIAI > 2 and AITL score > 2, respectively. Progression‐free survival (PFS) and OS were chosen as the endpoints to evaluate the prognoses of AITL patients. PFS is defined as the duration from diagnosis confirmation to first disease relapse, progression, death from all causes or last follow‐up. OS is the time from disease diagnosis to death from all causes or last follow‐up.

### 2.4 Statistical analysis

Continuous variables were expressed as mean ± standard deviation (SD) or median (interquartile range, IQR), and categorical variables were expressed as counts (percentages). Comparison analysis of different TMTV, Dmax levels and clinical parameters were tested by the Chi‐square test. The Mann‐Whitney *U* test was used to compare the differences in Dmax and TMTV between patients with stage III and IV. The optimal cut‐off values for TMTV and Dmax to determine prognosis were obtained by the receiver operator characteristic curves (ROCs) and the maximum Youden index method. Survival curves were plotted by the Kaplan–Meier method and log‐rank tests were performed. Cox proportional hazards regression models were used to assess the prognostic value of parameters. Univariate Cox analyses were used to find potential prognostic parameters. Parameters that were statistically significant in univariate analysis were included in multivariate Cox analysis models to explore independent prognostic values. Multiple multivariate Cox analysis models were built according to different comparison purposes and the principle of reducing collinearity. Combining parameters of independently prognostic value or clinical interest to assess the risk stratification ability. Statistical analyses were performed by SPSS 26.0 (IBM, USA) software and MedCalc Software (Ostend, Belgium), and two‐sided *p* < 0.05 was considered statistically significant.

## 3 RESULTS

### 3.1 Clinical characteristics

A total of 81 AITL patients (53 men and 28 women) were included, and the whole‐group clinical characteristics were shown in Table 1. The mean age was 63.1 ± 1.1 years, and the median age was 63 years. Over half of the patients had B symptoms, and 76 (93.8) cases were in III or IV stage at initial diagnosis. Forty‐five (55.6) patients had ENI, of which 13 (16.0) patients had ENIs ≥2. Bone marrow, skin, lung and gastrointestinal tract were the most common ENI sites. There were 50 (61.7) cases with splenomegaly, and 73 (90.0) cases were diagnosed with spleen involvement according to the above criteria in PET/CT.

**TABLE 1:** Clinical characteristics of the whole group

Characteristics	Counts (%)	Characteristics	Counts (%)
Age, mean ± SD (year)	63.1 ± 1.1	CRP	
Gender		Elevated	54 (66.7)
Female	28 (34.6)	Normal	17 (21.0)
Male	53 (65.4)	Unknown	10 (12.3)
B symptoms		ENIs	
Yes	50 (61.7)	≥2	13 (16.0)
No	31 (38.3)	<2	68 (84.0)
ECOG‐PS,		EBV‐DNA	
≥2	27 (33.3)	Positive	34 (42.0)
<2	54 (66.7)	Negative	43 (53.1)
Ann Arbor		Unknown	4 (4.9)
II	5 (6.2)	Treatment regimens	
III	37 (45.7)	CHOP	27 (33.3)
IV	39 (48.1)	CHOPE	46 (56.8)
BMI		Other	8 (9.9)
Yes	20 (24.7)	IPI	
No	61 (75.3)	0–2	33 (40.7)
Haemoglobin		3–5	48 (59.3)
Low	58 (71.6)	PIT	
normal	23 (28.4)	0–2	62 (76.5)
PLT counts		3–4	19 (23.5)
<150 × 10^9^/L	31 (38.3)	PIAI	
≥150 × 10^9^/L	50 (61.7)	0–2	54 (66.7)
LDH		3–5	27 (33.3)
Elevated	54 (66.7)	AITL score	
Normal	27 (33.3)	0–2	38 (46.9)
Albumin		3–4	33 (40.7)
Low	69 (85.2)	Unknown	10 (12.3)
Normal	12 (14.8)	TMTV, median (IQR) (cm^3^)	383.8 (139.5, 702.4)
β2‐MG		Dmax, median (IQR) (cm)	66.4 (58.4, 73.2)
Elevated	64 (79.0)		
Normal	17 (21.0)		

Abbreviations: AITL, angioimmunoblastic T‐cell lymphoma; BMI, bone marrow involvement; CHOP, cyclophosphamide, doxorubicin, vincristine, and prednisone; CHOPE, CHOP plus etoposide; CRP, C‐reactive protein; Dmax, the distance between the two lesions that are the furthest apart; EBV‐DNA, Epstein‐Barr virus DNA; ECOG‐PS, Eastern Cooperative Oncology Group performance status; ENIs, extranodal involvement sites; IPI, International Prognostic Index; IQR, interquartile range.; PIAI, Prognostic Index for AITL; PIT, Prognostic Index for T‐cell lymphoma; PLT, platelet; TMTV, total metabolic tumour volume; β2‐MG, β2‐microglobulin

### 3.2 Treatment and survival

The majority of this cohort received anthracycline‐based chemotherapy regimens, with 27 (33.3) cases receiving CHOP (cyclophosphamide, doxorubicin, vincristine and prednisone) regimen, 46 (56.8) cases receiving CHOPE (CHOP plus etoposide) regimen, and eight (9.9) cases treated by other regimens (gemcitabine plus oxaliplatin, three patients; chidamide plus cyclophosphamide plus prednisone, three patients; brentuximab vedotin plus cyclophosphamide, doxorubicin, and prednisone, two patients). After a median follow‐up time of 19.4 (IQR: 8.5–46.9) months, a total of 53 (65.4) patients experienced progression, including 43 (53.1) patients who died. Median PFS and OS were 10.5 (95% confidence interval [CI]: 5.1–15.9] months and 36.7 (95% CI: 10.8–62.6) months, respectively. The 1‐, 3‐ and 5‐year PFS rates were 47.5%, 33.3% and 28.4%, and the 1‐, 3‐ and 5‐year OS rates were 68.6%, 50.5% and 44.2%, respectively.

### 3.3 The cut‐off values of TMTV and Dmax by ROC curves

The median TMTV and Dmax are shown in Table 1. The cut‐off value of TMTV was 456.6 cm^3^ (area under the curve [AUC] = 0.67, 95% CI: 0.55–0.78, *p* = 0.010], and the sensitivity and specificity for OS were 60.5% and 73.7%, and 49.1% and 64.3% for PFS, respectively. The cut‐off value of Dmax was 65.7 cm (AUC = 0.67, 95% CI: 0.55–0.79, *p* = 0.011), and the sensitivity and specificity for OS were 74.4% and 63.2%, and 66.0% and 60.7% for PFS, respectively. The accuracy, positive predictive value and negative predictive value in predicting PFS and OS are listed in Table 2.

**TABLE 2:** Prediction of endpoints with TMTV and Dmax

	Progression‐free survival		Overall survival	
	TMTV	Dmax	TMTV	Dmax
Se (%)	49.1	66.0	60.5	74.4
Sp (%)	64.3	60.7	73.7	63.2
NPV (%)	40.0	51.4	62.2	68.6
PPV (%)	72.0	76.1	72.2	69.6
Acc (%)	54.3	64.2	66.7	69.1

Abbreviations: Acc, accuracy; Dmax, the distance between the two lesions that are the furthest apart.; NPV, negative predictive value; PPV, positive predictive value; Se, sensitivity; Sp, specificity; TMTV, total metabolic tumour volume

### 3.4 Comparison of patient clinical data with TMTV and Dmax

Patient characteristics and comparative analysis stratified according to high and low Dmax and TMTV values were shown in Table 3. From this table, high TMTV was associated with low haemoglobin, PLT counts and albumin, as well as elevated LDH, β2‐MG, CRP and EBV‐DNA levels. And also, patients with age >60, ECOG‐PS ≥ 2 and the advanced stage were usually accompanied by high TMTV. In terms of the four risk assessment models, high‐risk patients tend to have higher TMTV, which was also reflected in Dmax. In addition, Dmax was associated with gender, Ann Arbor stage, haemoglobin, PLT counts, LDH and β2‐MG levels. The proportion of patients with high TMTV and high Dmax was higher in patients with positive BMI. Besides, if the five patients in stage II were excluded, Dmax (median: 64.6 cm vs. 70.8 cm, *p* = 0.010) and TMTV (median: 327.4 cm^3^ vs. 592.8cm^3^, *p* = 0.001) in stage III and IV patients still showed statistical differences.

**TABLE 3:** Comparison of patient clinical data with TMTV and Dmax

Characteristics		TMTV			Dmax		
	All = 81	Low (*n* = 45)	High (*n* = 36)	*p‐*Value^a^	Low (*n* = 35)	High (*n* = 46)	*p*‐Value^b^
Age, ≤60/>60	32/49	24/21	8/28	0.004	14/21	18/28	0.937
Gender, female/male	28/53	16/29	12/24	0.834	17/18	11/35	0.021
B symptoms, yes/no	50/31	24/21	26/10	0.082	18/17	32/14	0.096
ECOG‐PS,≥2/<2	27/54	9/36	18/18	0.004	8/27	19/27	0.081
Ann Arbor, II/III/IV	5/37/39	5/26/14	0/11/25	0.001	5/21/9	0/16/30	<0.001
ENIs, ≥2/<2	13/68	5/40	8/28	0.176	3/32	10/36	0.110
BMI, yes/no	21/60	4/41	17/19	<0.001	3/32	18/28	0.002
Haemoglobin, low/normal	58/23	25/20	33/3	<0.001	18/17	40/6	<0.001
PLT,<150 × 10^9^/L/normal	31/50	9/36	22/14	<0.001	6/29	25/21	0.001
LDH, elevated/normal	54/27	24/21	30/6	0.004	19/16	35/11	0.039
Albumin, low/normal	69/12	34/11	35/1	0.006	28/7	41/5	0.252
β2‐MG, elevated/normal	64/17	28/17	36/0	<0.001	22/13	42/4	0.002
CRP, elevated/normal	54/17	25/13	29/4	0.030	19/10	35/7	0.084
EBV‐DNA, positive/negative	33/43	13/31	21/12	0.003	13/21	21/22	0.352
IPI, 0‐2/3‐5	33/48	27/18	6/30	<0.001	19/16	14/32	0.030
PIT, 0‐2/3‐4	55/26	38/7	17/19	<0.001	32/3	30/16	0.006
PIAI, 0‐2/3‐5	54/27	37/8	17/19	0.001	30/5	24/22	0.002
AITL score, 0‐2/3‐4	33/38	27/11	11/22	0.001	20/9	18/24	0.030
TMTV, high/low	36/45	–	–	–	9/26	27/19	0.003

Abbreviations: AITL, Angioimmunoblastic T‐cell lymphoma; BMI, bone marrow involvement; CRP, C‐reactive protein; Dmax, the distance between the two lesions that are the furthest apart.; EBV‐DNA, Epstein‐Barr virus DNA; ECOG‐PS, Eastern Cooperative Oncology Group performance status; ENIs, extranodal involvement sites; IPI, International Prognostic Index; PIAI, Prognostic Index for AITL; PIT, Prognostic Index for T‐cell lymphoma; PLT, platelet; TMTV, total metabolic tumour volume; β2‐MG, β2‐microglobulin

^a^ *p*‐Value of the chi‐square test between different TMTV levels and clinical characteristics.

^b^ *p*‐Value of the chi‐square test between different Dmax levels and clinical characteristics.

### 3.5 Univariate survival analysis

Univariate Cox survival analysis is shown in Table 4. For PFS, elevated CRP, positive BMI, PLT counts < 150 × 10^9^/L, PIT > 2, PIAI > 2, AITL score > 2, high TMTV and high Dmax were all prognostic risk factors. For OS, ECOG‐PS ≥ 2, positive BMI, PLT counts < 150 × 10^9^/L, elevated β2‐MG, IPI > 2, PIT > 2, PIAI > 2, AITL score > 2, high TMTV and high Dmax were all risk factors for OS.

**TABLE 4:** Univariate survival analysis

	Progression‐free survival		Overall survival	
Characteristic	HR (95% CI)	*p‐*Value	HR (95% CI)	*p‐*Value
Age, ≤60(ref)/>60	1.23 (0.71–2.13)	0.466	1.57 (0.83–2.95)	0.165
Gender, female(ref)/male	1.05 (0.60–1.84)	0.862	1.09 (0.58–2.05)	0.788
B symptoms, no(ref)/yes	1.22 (0.69–2.15)	0.491	1.46 (0.76–2.83)	0.346
ECOG‐PS, <2(ref)/≥2	1.53 (0.88–2.66)	0.123	2.16 (1.19–3.95)	0.012
Ann Arbor, II(ref)/III‐IV	0.83 (0.26–2.68)	0.760	22.62 (0.12–4375.46)	0.246
ENIs, <2(ref)/≥2	1.85 (0.97–3.52)	0.063	1.69(0.83–3.45)	0.147
BMI, no(ref)/yes	1.92 (1.07–3.43)	0.029	2.66 (1.41–5.00)	0.002
Haemoglobin, normal(ref)/low	1.35 (0.72–2.52)	0.351	1.90 (0.88–4.10)	0.101
PLT, normal(ref)/<150×10^9^/L	1.92 (1.11–3.31)	0.019	2.37 (1.30–4.34)	0.005
LDH, normal(ref)/elevated	0.95 (0.55–1.67)	0.850	1.59 (0.80–3.16)	0.184
Albumin, normal(ref)/low	0.83 (0.40–1.69)	0.599	2.11 (0.79–5.67)	0.138
β2‐MG, normal(ref)/elevated	1.84 (0.86–3.90)	0.114	3.32 (1.18–9.33)	0.023
CRP, normal(ref)/elevated	2.49 (1.16–5.35)	0.019	1.98 (0.87–4.50)	0.102
EBV‐DNA, negative(ref)/positive	0.70 (0.39–1.24)	0.220	0.52 (0.81–1.54)	0.520
IPI, 0‐2(ref)/3‐5	1.28 (0.74–2.22)	0.374	2.14 (1.13–4.08)	0.020
PIT, 0‐2(ref)/3‐4	2.14 (1.18–3.88)	0.012	2.62 (1.40–4.89)	0.003
PIAI, 0‐2(ref)/3‐5	1.79 (1.03–3.12)	0.038	2.42 (1.33–4.42)	0.004
AITL score, 0‐2(ref)/3‐4	2.19 (1.23–3.87)	0.007	2.52 (1.34–4.76)	0.004
TMTV, ≤456.6 cm^3^(ref)/>456.6 cm^3^	1.74 (1.01–3.00)	0.046	2.86 (1.55–5.29)	0.001
Dmax, ≤65.7 cm(ref)/ > 65.7 cm	1.98 (1.12–3.51)	0.020	2.74 (1.38–5.46)	0.004

Abbreviations: AITL, Angioimmunoblastic T‐cell lymphoma; BMI, bone marrow involvement; CI, confidence interval; CRP, C‐reactive protein; Dmax, the distance between the two lesions that are the furthest apart; EBV‐DNA, Epstein‐Barr virus DNA; ECOG‐PS, Eastern Cooperative Oncology Group performance status; ENIs, extranodal involvement sites; HR, hazard ratio; IPI, International Prognostic Index; PIAI, Prognostic Index for AITL; PIT, Prognostic Index for T‐cell lymphoma; PLT, platelet; ref, reference.; TMTV, total metabolic tumour volume; β2‐MG, β2‐microglobulin

**FIGURE 2:**
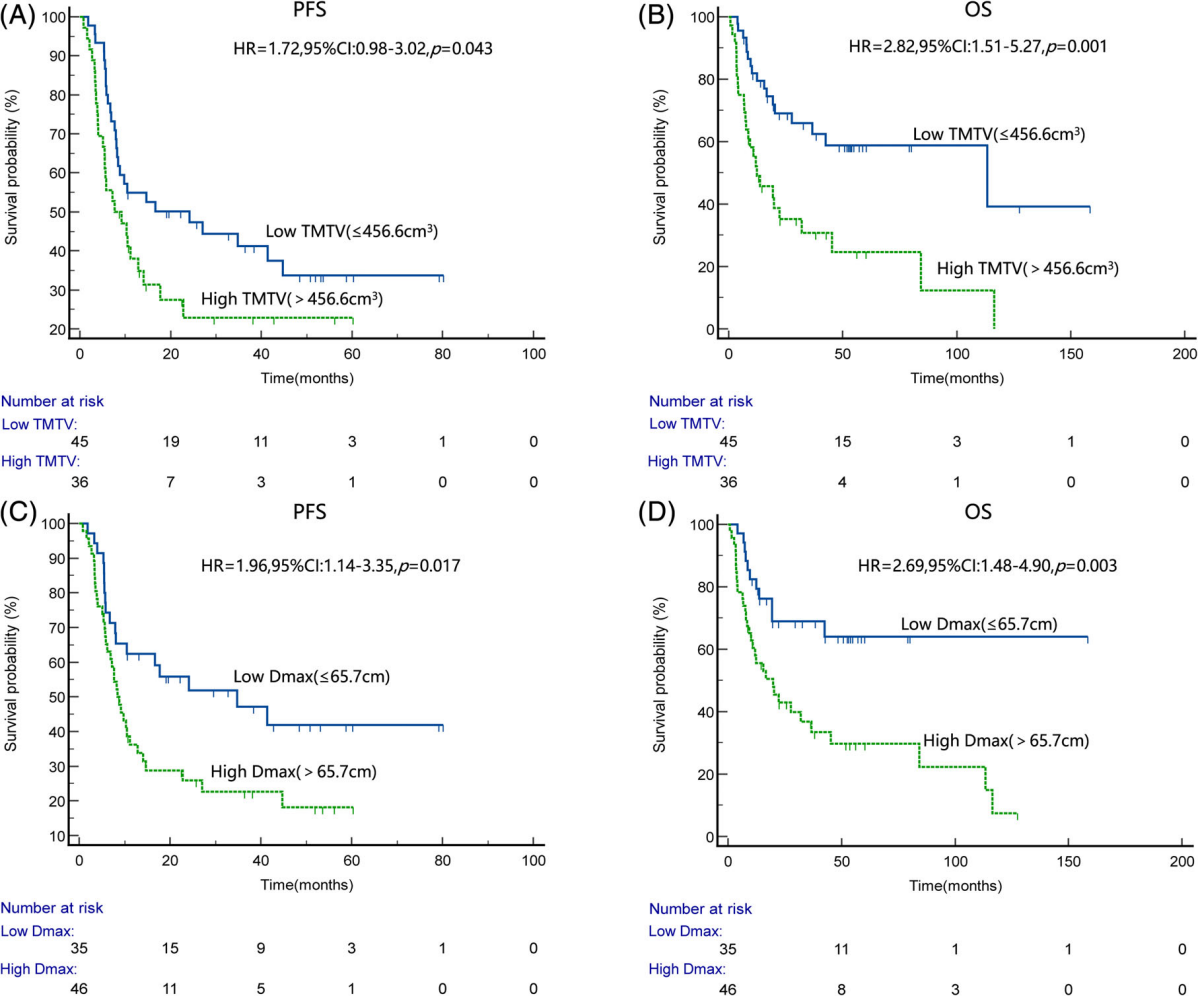
Kaplan–Meier survival curves and log‐rank test for progression‐free survival (PFS) and overall survival (OS) of the whole group of patients according to different levels of total metabolic tumour volume (TMTV) or Dmax. (A and B) PFS and OS curves according to TMTV, respectively. (C and D) PFS and OS curves according to Dmax, respectively

Kaplan‐Meier survival curves (Figure 2) and log‐rank tests for TMTV and Dmax showed that high TMTV (>456.6 cm^3^) and high Dmax (>65.7 cm) were associated with poorer PFS and OS. Patients with high TMTV had significantly lower PFS rates (3‐year PFS rate: 22.9% vs. 41.2%) and OS rates (3‐year OS rate: 30.8% vs. 65.9%) than patients with low TMTV (≤456.6 cm^3^). Similarly, PFS rates (3‐year PFS rate: 22.7% vs. 47.1%) and OS rates (3‐year OS rate: 36.8% vs. 68.9%) in high Dmax patients were lower significantly than in low Dmax patients (≤65.7 cm).

There was no significant difference in PFS rate (*p* = 0.526) and OS rate (*p* = 0.885) between patients receiving CHOP and CHOPE regimens. In the subgroup analysis, there was no significant difference in PFS and OS rate between stage III and IV patients (3‐year PFS rate: 42.3% vs. 26.3%; *p* = 0.136; 3‐year OS rate: 53.0% vs. 43.5%, *p* = 0.290), while survival rates remained statistically different between patients with low or high Dmax (3‐year PFS rate: 49.2% vs. 22.7%, *p* = 0.002; 3‐year OS rate: 64.8% vs. 36.8%, *p* = 0.010)

### 3.6 Multivariate survival analysis

To investigate the independent prognostic value of PET parameters, six multivariate Cox analysis models were established (Table 5). Four of these models were suitable for PFS. Only TMTV and Dmax were included in model 1, and the results showed that high Dmax (>65.7 cm) could be an independent risk factor for PFS (HR = 1.98, 95% CI: 1.12–3.51*, p* = 0.020), while TMTV was not significant. From model 2 to model 4, PIT, PIAI and AITL score were introduced in the multivariate analysis with TMTV and Dmax, respectively. The results showed that PIT > 2 (HR = 2.14, 95%CI: 1.18–3.88, *p* = 0.012) and AITL score > 2 (HR = 2.19, 95%CI: 1.23–3.87, *p* = 0.007) could be used as independent risk factors for PFS, and Dmax (HR = 1.98, 95% CI: 1.12–3.51, *p* = 0.02) demonstrated prognostic value independent of PIAI. Model 6 included all the individual factors that were significant in univariate analysis. Elevated CRP (HR = 1.88, 95% CI: 1.01–3.51, *p* = 0.047) and high Dmax (HR = 2.19, *p* = 0.048) were independent prognostic factors for PFS. In model 1 to model 6, both TMTV and Dmax remained independent prognostic factors for OS (in models 1 to 4, model 6, TMTV: HR = 2.31, 95% CI: 1.22–4.38, *p* = 0.010; Dmax: HR = 2.13, 95% CI: 1.04–4.36, *p* = 0.040; in model 5, TMTV: HR = 2.37, 95% CI: 1.21–4.61, *p* = 0.011, Dmax: HR = 2.14, 95% CI: 1.02–4.52, *p* = 0.045).

**TABLE 5:** Multivariate survival analysis

	Progression‐free survival		Overall survival	
Characteristics	HR (95% CI)	*p*‐Value	HR (95% CI)	*p*‐Value
**Model 1**				
TMTV>456.6 cm^3^	NS	NS	2.31(1.22–4.38)	0.010
Dmax>65.7 cm	1.98 (1.12–3.51)	0.020	2.13 (1.04–4.36)	0.040
**Model 2**				
TMTV>456.6 cm^3^	NS	NS	2.31(1.22–4.38)	0.010
Dmax>65.7 cm	NS	NS	2.13 (1.04–4.36)	0.040
PIT,3‐4	2.14 (1.18–3.88)	0.012	NS	NS
**Model 3**				
TMTV>456.6 cm^3^	NS	NS	2.37 (1.21–4.61)	0.011
Dmax>65.7 cm	NS	NS	2.14 (1.02–4.52)	0.045
AITL score,3‐4	2.19 (1.23–3.87)	0.007	NS	NS
**Model 4**				
TMTV>456.6 cm^3^	—	—	2.31 (1.22–4.38)	0.010
Dmax>65.7 cm	1.98 (1.12–3.51)	0.020	2.13 (1.04–4.36)	0.040
PIAI,3‐5	—	—	NS	NS
**Model 5**				
TMTV>456.6 cm^3^	—	—	2.31 (1.22–4.38)	0.010
Dmax>65.7 cm	—	—	2.13 (1.04–4.36)	0.040
IPI,3‐5	—	—	NS	NS
**Model 6**				
TMTV>456.6 cm^3^	NS	NS	2.31 (1.22–4.38)	0.010
Dmax>65.7 cm	2.19 (1.01–4.74)	0.048	2.13 (1.04–4.36)	0.040
ECOG‐PS,2‐5	—	—	NS	NS
BMI, yes	NS	NS	NS	NS
PLT<150 × 10^9^/L	NS	NS	NS	NS
Elevated β2‐MG	—	—	NS	NS
Elevated CRP	1.88 (1.01–3.51)	0.047	—	—

Abbreviations: AITL, angioimmunoblastic T‐cell lymphoma; BMI, bone marrow involvement; CI, confidence interval; CRP, C‐reactive protein; Dmax, the distance between the two lesions that are the furthest apart; ECOG‐PS, Eastern Cooperative Oncology Group performance status; HR, hazard ratio; IPI, International Prognostic Index; NS, no significance.; PIAI, Prognostic Index for AITL; PIT, Prognostic Index for T‐cell lymphoma; PLT, platelet; TMTV, total metabolic tumour volume; β2‐MG, β2‐microglobulin

### 3.7 Risk stratification

**FIGURE 3:**
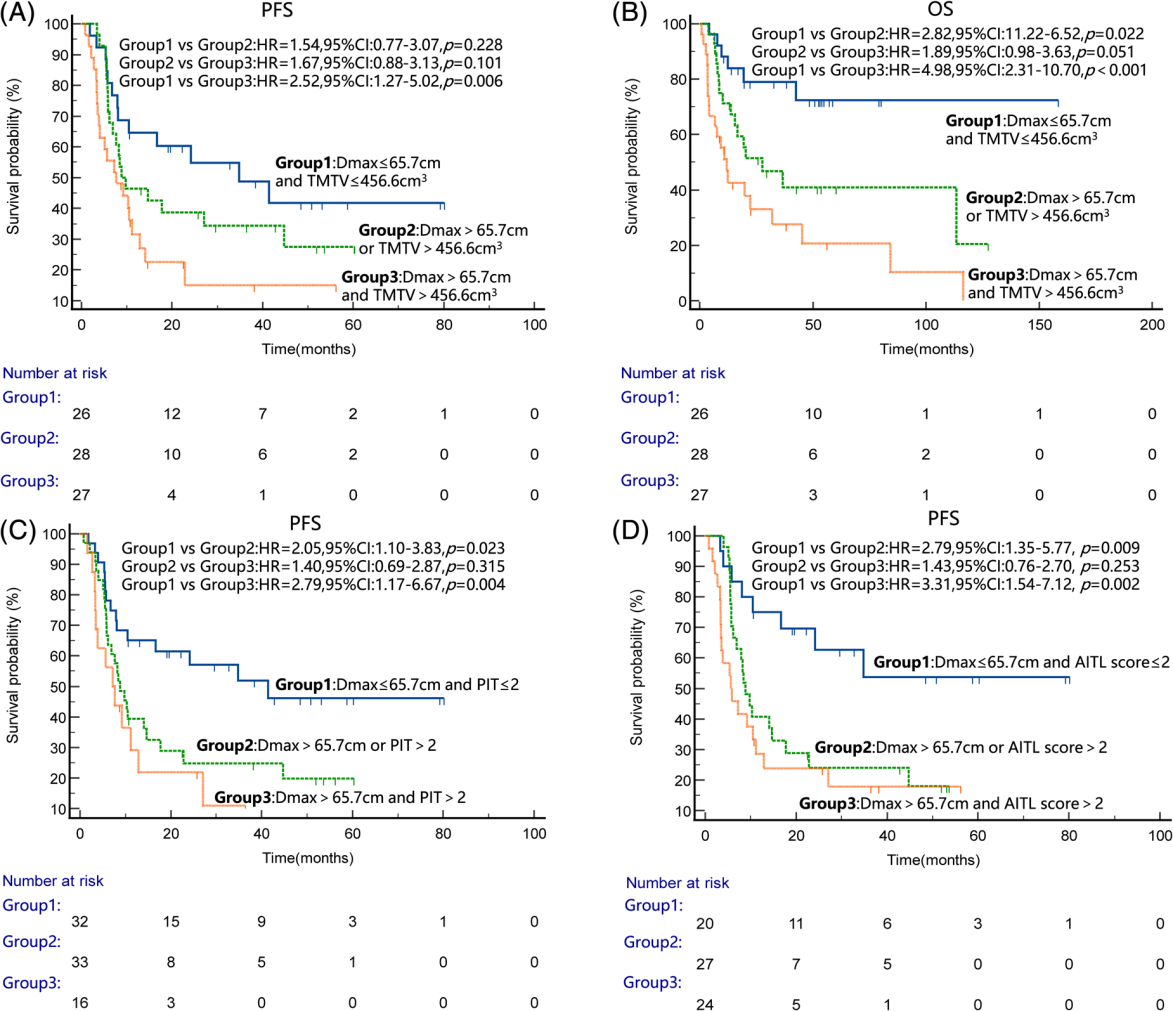
Kaplan–Meier survival curves for the whole group of patients according to different models. (A and B) Progression‐free survival (PFS) and overall survival (OS) survival curves according to total metabolic tumour volume (TMTV) combined with Dmax, respectively. (C and D) PFS survival curves according to Dmax combined with Prognostic Index for T‐cell lymphoma (PIT) or angioimmunoblastic T‐cell lymphoma (AITL) score, respectively

**FIGURE 4:**
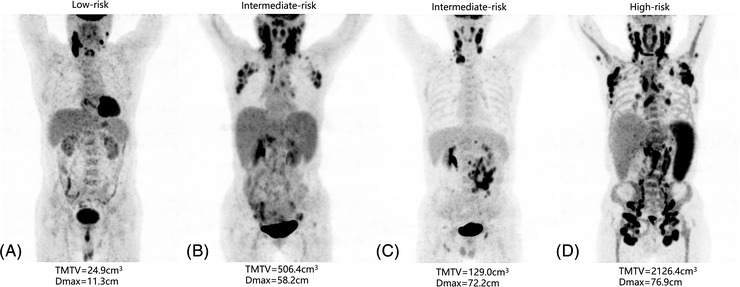
Examples of fluoro‐D‐glucose (FDG) positron emission tomography (PET) images (maximum‐intensity projections) of patients in different risk groups. (A) Low total metabolic tumour volume (TMTV) and low Dmax; (B) high TMTV and low Dmax; (C) low TMTV and high Dmax; (D) high TMTV and high Dmax

According to multivariate analysis results, three risk categories could be distinguished based on Dmax and TMTV. Patients with high TMTV and high Dmax, high TMTV or high Dmax, and low TMTV and low Dmax were defined separately as the high‐risk group (Group 1), intermediate‐risk group (Group 2), and low‐risk group (Group 3). Finally, there were 27 (33.3), 28 (34.6) and 26 (32.1) cases in the high‐risk, intermediate‐risk, and low‐risk group, respectively. The 3‐year PFS rates were 48.7%, 34.4% and 15.0% for the high‐risk, intermediate‐risk, and low‐risk groups, respectively (Group 1 vs. Group 2, HR = 1.54, *p* = 0.228; Group 1 vs. Group 3, HR = 2.52, *p* = 0.006; Group 2 vs. Group 3, HR = 1.67, *p* = 0.101), and the 3‐year OS rates were 79.0%, 46.8% and 27.6%, respectively (Group 1 vs. Group 2, HR = 2.82, *p* = 0.022; Group 1 vs. Group 3, HR = 4.98, *p* < 0.001; Group 2 vs. Group 3, HR = 1.89, *p* = 0.051) (Figure 3A,B). In the intermediate‐risk group, patients with low Dmax and high TMTV had eight cases and 20 cases with high Dmax and low TMTV, both had similar PFS rates (*p* = 0.76) and OS rates (*p* = 0.33). Figure 4 shows examples of PET images (maximum‐intensity projections) of patients in different risk groups.

Furthermore, Dmax also showed an independent prognostic value for PFS, so we compared the prognostic value of Dmax combined with PIT or AITL score for PFS, respectively and grouped them as described above. In the Dmax combined with PIT model, the 3‐year PFS rates were 51.9% and 24.8% for patients in the low‐risk (Group 1) and intermediate‐risk (Group 2) (Group 1 vs. Group 2, HR = 2.05, *p* = 0.023). In the Dmax combined with AITL score model, the 3‐year PFS rates for patients in the low‐risk (Group 1) and intermediate‐risk (Group 2) were 53.7% and 24.0% (Group 1 vs. Group 2:HR = 2.70, *p* = 0.009) (Figure 3C,D). The combinations of Dmax and PIT or AITL could improve the ability to distinguish low‐risk from intermediate‐risk patients for PFS.

## 4 DISCUSSION

The prognosis of AITL is poor and heterogeneous. Early identification of high‐risk patients unlikely to be cured by conventional frontline regimens is a key step for alternative treatments. This requires a reliable risk stratification model. Parameters extracted from baseline PET can play an important role for FDG‐avid AITL patients [8, 19]. Due to the low prevalence, few independent cohorts validated the prognostic value of TMTV in AITL. The small sample with 56 AITL patients from our centre showed that high baseline TMTV could independently predict the PFS and OS [8]. In the present study, we validated preliminarily the prognostic value of PET parameter Dmax in AITL. Furthermore, Dmax combined with TMTV could identify high‐risk AITL patients before treatment.

The prognostic value of Dmax has been established initially in DLBCL and HL. Cottereau et al. retrospectively included 95 patients with stage III/IV DLBCL patients with at least two positive lesions in baseline PET [12]. Their study showed that high Dmax (>58 cm) was an independent prognostic factor for PFS, while high TMTV (>394cm^3^) was not. For OS, both TMTV and Dmax were independent predictors. After this study, they investigated the prognostic value of SDmax (Dmax standardized by body surface area) in a larger cohort including 290 DLBCL patients. The results also showed that SDmax (>0.32 m^−1^) combined with TMTV (>220cm^3^) could be used for risk stratification independently of IPI, providing a promising new and concise model for prognostic stratification of DLBCL patients [14].

Studies of various cancers have shown different degrees of molecular heterogeneity within and between biopsy specimens from different sites in the same patient, which emphasize the importance of spatial heterogeneity [20]. Radiomics analysis is expected to reflect heterogeneity within the lesion or between lesions from the imaging aspect. Current textural and morphological analyses of FDG PET tumour metabolic patterns have shown the ability to enhance prognostic prediction in lymphoma, while those studies mainly focused on B‐cell lymphoma and HL, and little evidence could provide extra diagnostic or prognostic information [15, 21, 22]. Moreover, texture parameters are mostly calculated from selected tumoral lesions. Lymphoma usually spreads throughout the body, especially AITL, and texture analysis of only partial lesions may be insufficient to characterize the heterogeneity features of disease [23, 24]. As a quantitative PET parameter, TMTV can describe the systemic tumour burden and include all or most lymphoma lesions, which is consistent with the importance of considering the spatial heterogeneity of the disease.

Dmax may reflect tumour heterogeneity in another dimension, which visualizes directly the ability of tumor dissemination and is superior to the traditional Ann Arbor staging. No significant difference was found in PFS and OS rates between stage III and stage IV patients with limited risk‐stratification ability. Dmax remained statistically different between those patients, suggesting powerful discriminative ability.

Dmax is a relatively simple 3D dissemination PET parameter, which can intuitively capture the patient‐based spatial migration feature of the disease. This metric is different from complex radiological features that are usually difficult to explain from a biological perspective. It is defined as the distance between the centroids of two lesions thus not to be highly affected by PET/CT scanner performance and scanning protocols, with widespread application. Moreover, MTV measurements may not have a substantial impact on Dmax because rare variations were obviously observed in the centroid of the lesion with the different lesion sizes. A comparative methodological study on TMTV and Dmax measurement pointed out that although the results of Dmax calculated based on different threshold methods differ mildly, the prognostic value is not significantly affected, suggesting that Dmax has good prognostic robustness [25].

In the process of performing the multivariate analysis, we established six models by different combinations, and the results showed that Dmax had independent prognostic values both for OS and PFS, while TMTV was significant only for OS. The model based on TMTV and Dmax could effectively differentiate three different risk groups and was most significant for OS. That is, the concise PET model can identify a group of high‐risk patients with poor prognosis, for whom clinicians may also consider consolidation with other treatments, such as autologous stem cell transplantation [26]. Dmax remained an independent prognostic value for PFS, the combination of Dmax with PIAI or AITL score showed a slightly stronger efficiency of risk stratification for PFS than the combination of Dmax with TMTV, especially for patients in low‐risk and intermediate‐risk groups. The above results support the satisfactory prognostic value of Dmax. In contrast, for PFS, TMTV was not significant, which may be partly explained by the cut‐off values selection and the limitation of statistical power.

However, Dmax‐ and TMTV‐based score have to be correlated with clinical or biological data and validated in larger cohorts for the purpose of guiding clinical practice. AITL score is the latest prognostic model established by four clinical parameters in a larger cohort of AITL and is considered to reflect the disease characteristics of AITL [5]. The present study showed that elevated CRP, which is included in the AITL score, could be an independent risk factor for PFS, but OS was not significant. This may be related to the lack of baseline CRP data in some patients, but it also confirms to some extent the independent prognostic value of CRP. Baseline low PLT, elevated β2‐MG, poorer performance status, positive EBV‐DNA and elderly patients may serve as clinical or biological parameters to complement TMTV or Dmax, but the current sample of AITL‐based studies was mostly small, and it is difficult to draw consistent conclusions between different studies. Large studies are needed to determine the best combination of prognostic factors and the factors most readily used in clinical practice. With the mature application of next‐generation sequencing technology, the therapeutic value of circulating tumour DNA testing is gradually emerging, and PET parameters combined with genetic and molecular profiling is one of the future research directions [27]. Durmo et al. showed that the differences in Dmax were mainly related to changes in the expression of the microenvironmental components by radiogenomics analysis and supported Dmax as a new prognostic factor for iPET‐negative classic HL patients with frontline treatment [16]. Because the calculation method has not been standardized, the clinical application of MTV is limited. Nevertheless, standardization of MTV calculation is underway and will soon be achieved at the international level [28]. Although Dmax is in its early stages, our results strongly suggest that current prognostic models can be further refined by including dissemination parameters derived from PET imaging.

This study had the following limitations. First, this study was limited to its retrospective nature. Second, the sample size was small, and the time span was wide. PET was an optional examination in the early period, and some patients lack baseline PET. Moreover, not all AITL lesions were FDG‐avid, and patients with low or no uptake were excluded from the study, which may cause selection bias. Lastly, a high proportion of splenomegaly and diffuse bone marrow uptake patients in this cohort made it difficult to accurately determine lymphoma infiltration, which may cause tumour burden overestimation.

## 5 CONCLUSIONS

As a tumour dissemination parameter, Dmax complements the prognostic value of TMTV in AITL patients. A concise model based on TMTV and Dmax may improve the prognostic value of PET staging and is expected to guide individualized treatment. These results need to be further evaluated in other large cohorts and compared with existing prognostic models to overcome the limitations of current clinical prognostic indicators in AITL patients.

## CONFLICT OF INTEREST

The authors declare that there is no conflict of interest that could be perceived as prejudicing the impartiality of the research reported.

## FUNDING INFORMATION

There was no funding support for this research.

## AUTHOR CONTRIBUTIONS

Lijun Tang and Chongyang Ding contributed as corresponding authors. Huanyu Gong and Bo Tang contributed equally to this article. Huanyu Gong and Bo Tang participated in the design of the study, carried out analysis and interpretation of data and drafted the manuscript; finally approved the version to be published and agreed to be accountable for all aspects of the work. Huanyu Gong, Tiannv Li and Jianyong Li participated in image analysis and in the discussion of the result of the part and final approval of the version to be published. Chongyang Ding and Lijun Tang gave the conception and design of the study, participated in the image analysis and discussion of the analysis of the results and approved the final submission. All authors read and approved the final manuscript.

## ETHICS STATEMENT

All informed consent forms were waived because this study was retrospective. This study was approved by the Ethics Committee of Jiangsu Province Hospital, the First Affiliated Hospital of Nanjing Medical University.

## Supporting information


**Table S1**. Four clinical risk assessment models

## Data Availability

Data are available upon reasonable request to the corresponding author.
