# Supplementary material for: The added prognostic values of baseline PET dissemination parameter in patients with angioimmunoblastic T‐cell lymphoma
Source: EJHaem. 2022 Nov 29;4(1):67–77. doi: 10.1002/jha2.610 (PMC9928789; doi:10.1002/jha2.610)
Supplement: Supplementary file 1 — Table S1. Four clinical risk assessment models [file JHA2-4-67-s001.docx]

**Supplement**

**Table 1.** Four clinical risk assessment models

| Risk models | Parameters |
| --- | --- |
| IPI | ①Age>60;②Ann Arbor stage III/IV;③Elevated LDH;④ECOG-PS≥2;⑤ENIs≥2 |
| PIT | ①Age>60;②Elevated LDH;③ECOG-PS≥2;④BMI |
| PIAI | ①Age>60;②ECOG-PS≥2;③ENIs≥2;④B symptoms;⑤PLT count <150×10^9^/L |
| AITL score | ①Age≥60;②ECOG-PS>2;③Elevated CRP;④Elevated β2-MG |

Abbreviations: ECOG-PS: Eastern Cooperative Oncology Group performance status, BMI: bone marrow involvement, PLT: platelet, β2-MG: β2-microglobulin, CRP: C-reactive protein, ENIs: extranodal involvement sites, IPI: International Prognostic Index, PIT: Prognostic Index for T-cell lymphoma, PIAI: Prognostic Index for AITL, AITL: Angioimmunoblastic T-cell lymphoma
